# Causal relationships between serum metabolites and coronary heart disease risk: a mendelian randomization study

**DOI:** 10.3389/fgene.2025.1440364

**Published:** 2025-03-20

**Authors:** Xiao-Yan Meng, Yong-Qing Zhu, Ying-Jie Zhang, Wei Sun, Shu-Ang Li

**Affiliations:** ^1^ Clinical Systems Biology Laboratories, The First Affiliated Hospital of Zhengzhou University, Zhengzhou, China; ^2^ The Academy of Medical Sciences, Zhengzhou University, Zhengzhou, China; ^3^ Department of Burn and Repair Reconstruction, The First Affiliated Hospital of Zhengzhou University, Zhengzhou, China

**Keywords:** coronary heart disease, serum metabolites, mendelian randomization, causal relationship, hexadecanedioate, risk factors

## Abstract

**Background:**

Coronary heart disease (CHD) represents a substantial global burden in terms of morbidity and mortality. Understanding the causal relationships between serum metabolites and CHD can provide a crucial understanding of disease mechanisms and potential therapeutic targets.

**Methods:**

We conducted a Mendelian randomization (MR) approach to explore the potential causal associations between serum metabolites and CHD risk. The primary analysis employed the inverse variance weighted (IVW) method, supplemented by additional analyses, including MR-Egger, weighted median, weighted mode, and sample mode. To bolster the robustness and reliability of our findings, we performed sensitivity analyses, which included evaluating, horizontal pleiotropy and leave-one-out analysis. Additionally, pathway enrichment analysis was conducted.

**Results:**

We identified 15 known and 11 unknown metabolites with potential associations to CHD. Among the known, six displayed protective effects, while nine were identified as risk factors. Notably, many of these metabolites are closely related to mitochondrial function, which was further supported by pathways and enrichment analysis. Using multiple statistical models to ensure robust results, we unveiled a significant association between hexadecanedioate, a palmitoyl lipid metabolized in mitochondria, and a ∼18% reduced risk of CHD (OR = 0.82, 95%CI: 0.72–0.93).

**Conclusion:**

MR analysis revealed 6 protective molecules, 9 hazardous metabolites associated with CHD. Many of these known metabolites are closely link to mitochondrial function, suggesting a critical role of mitochondria in CHD development. In particular, hexadecanedioate, an essential component for mitochondrial energy production, was inversely associated with CHD risk. This suggests that mitochondrial function, and specifically the role of hexadecanedioate, may be pivotal in the development and progression of CHD.

## Introduction

Coronary heart disease (CHD) stands as a prevalent cardiovascular disease characterized by the narrowing of coronary arteries, resulting in reduced blood flow to the heart muscle (Henderson, 1996). It continues to be a leading cause of illness and death worldwide, imposing a substantial burden on healthcare systems and individuals (Goff et al., 2021). While traditional risk factors such as age, hypertension, dyslipidemia, and tobacco use have been extensively studied, there exists a growing interest in understanding the role of novel biomarkers and metabolic pathways in the pathogenesis of CHD (Zhu and Li, 2023).

Serum metabolites link genetic variations to disease outcomes through metabolic pathways (Li et al., 2020; Vasishta et al., 2022). Quantifying and profiling these metabolites using metabolomics techniques hold great promise in identifying biomarkers and uncovering disrupted metabolic pathways in individuals with CHD (Talmor-Barkan et al., 2022). However, establishing causal relationships in this context is challenging (Talmor-Barkan et al., 2022). Nonetheless, exploring the causal associations between serum metabolites and CHD can provide valuable insights into identify potential therapeutic targets.

Mendelian randomization (MR), a method utilizing genetic variants as instrumental variables (Emdin et al., 2017), provides a valuable approach to establishing causal relationships in observational studies (Sekula et al., 2016). By capitalizing on the random assortment of genetic variants during gamete formation, MR provides unbiased estimates of the causal effects between exposures and outcomes (Zuber et al., 2022). In this study, serum metabolites are the exposure factors, and CHD is the outcome, MR serves as a tool to determine whether alterations in metabolite levels directly contribute to CHD development or act as mere bystanders (Borges et al., 2022).

In this study, we aim to employ the Mendelian randomization framework to investigate potential causal associations between serum metabolites and CHD risk. To achieve this, we will leverage extensive genome-wide association studies (GWAS) and genetic instruments—single nucleotide polymorphisms (SNPs) strongly associated with specific metabolite levels (Tsepilov et al., 2015; Mirkov et al., 2012). We seek to unravel the causal role of specific metabolites in CHD development. Through the application of MR, we will assess whether genetically determined differences in metabolite levels are associated with changes in CHD risk (Sekula et al., 2016). Attaining a comprehensive understanding of the causal connections between serum metabolites and CHD holds immense potential for advancing our knowledge of disease mechanisms, identifying novel therapeutic targets, and developing personalized prevention strategies.

## Materials and methods

### Study Design

To explore the potential causal relationship between serum metabolites and CHD, a two-sample MR study was undertaken. This investigation relies on three fundamental assumptions (Emdin et al., 2017). Firstly, the instrumental variables (IVs) must demonstrate a direct and highly significant association with the exposure, which in this case is serum metabolites. Secondly, there should be no connections between the IVs and the confounding factors, thereby ensuring unbiased estimates. Lastly, the IVs solely influence the outcome through the exposure, without any involvement of alternative pathways.

### Data resources

**FIGURE 1:**
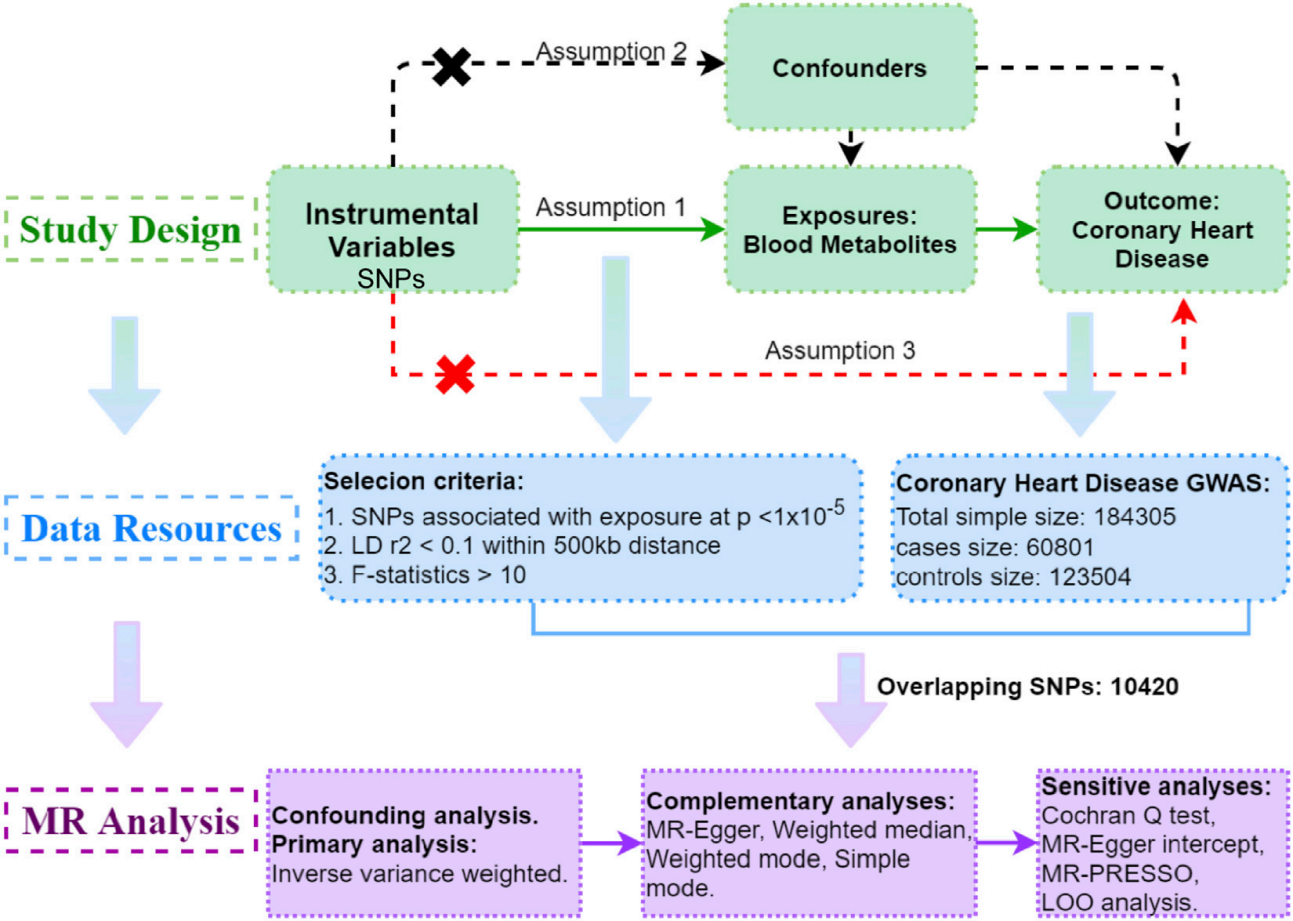
Study Design: Schematic overview of Two-Sample Mendelian Randomization Analyses in this study.

The data utilized in this study all came from the publicly available datasets, accessible on the database website, and have previously obtained ethics approval in the respective studies. The study’s progression is depicted in Figure 1.

The serum metabolite data employed in the GWAS analysis were sourced from Metabolomics GWAS server (https://metabolomics.helmholtz-muenchen.de/gwas/). The metabolomic database used was acquired from a seminal and comprehensive investigation conducted by Shin et al. (2014). The GWAS cohort encompassed 7,824 adult individuals of European descent, from whom genetic samples were obtained (Shin et al., 2014). The subsequent analysis involved an extensive examination of over 2.1 million SNPs within this population. Following meticulous quality control procedures, a total of 486 metabolites were deemed suitable for inclusion in the GWAS analysis. These metabolites were further categorized into 309 known metabolites and 177 metabolites with yet undetermined identification. The 309 known metabolites were subsequently classified into eight distinct biochemical groups, including amino acids, peptides, lipids, cofactors and vitamins, carbohydrates, energy-related compounds, nucleotides, and exotic substances.

The CHD data was obtained from the GWAS platform of the IEU OpenGWAS project (https://gwas.mrcieu.ac.uk/), with the dataset identifier assigned as ieu-a-7. We utilized the summary statistics from the existing meta-analysis of GWAS on CHD, combining data from 48 different studies. The analysis encompassed a total of 60,801 cases and 123,504 controls. Among the participants, the majority, approximately 77% had European ancestry, 13% had South Asian ancestry (from India and Pakistan), 6% had East Asian ancestry (from China and Korea), and there were smaller samples represented Hispanic and African Americans.

### Select instrumental variable

The criteria for selecting instrumental variables in Mendelian randomization need to satisfy the following assumptions.

(1) Relevance: The IVs must be strongly correlated with the exposure (coronary heart disease).
(2) Independence: The IVs should not be associated with any confounders.
(3) Exclusion Restriction: The IVs should only affect the outcome through the exposure.

To satisfy assumption (1), a rigorous screening process was conducted to identify IVs associated with blood metabolites. Considering the limited number of metabolite-associated SNPs, a slightly relaxed significance threshold of *p* < 1 × 10^−5^ was adopted to select relevant SNPs (Sanna et al., 2019). Subsequently, SNPs were grouped by removing linkage disequilibrium (LD) using a threshold of R^2^ > 0.1 and within a distance of 500 kb. To mitigate potential bias from weak instruments, each SNP underwent R^2^ (Formula 1) and F (Formula 2) statistic calculations based on various parameters such as effect size (β), standard error (SE), effect allele frequency (EAF), instrumental variable (R^2^), and sample size (N) (Burgess et al., 2011). SNPs with an F statistic < 10 were considered inadequate instruments and excluded from further analysis.

Formula 1:

$$R^{2}=\frac{2\times \beta^{2\;}\times EAF\times \left(1-EAF\right)}{\left[2\times \beta^{2\;}\times EAF\times \left(1-EAF\right)+2\times {SE}^{2}\times N\times EAF\times \left(1-EAF\right)\right]}$$ (1)

Formula 2:

$$F=\frac{R^{2\;}\times \left(N‐2\right)}{\left(1‐R^{2}\right)}$$ (2)

Subsequently, metabolite-associated SNPs were extracted from the outcome. Harmonization was applied to ensure consistency between exposure and outcome variables by aligning the effect alleles across the datasets. This was done by checking the direction of effect for each SNP, ensuring that the exposure and outcome variables were coded consistently, and resolving any discrepancies in allele orientation. To meet assumption (3), outcome-related SNPs (*p* < 1 × 10^−5^) within the IVs were also removed. Finally, a robust MR analysis was performed exclusively on metabolites that possessed more than two SNPs, guaranteeing a thorough investigation of causal relationships (Figure 1).

### MR analysis

In this study, we employed the IVW model as the primary two-sample MR analysis approach (Dudbridge, 2021). This model relies on key assumptions, including the relevance, independence, and exclusivity of IVs, as well as the notion that genetic variations impact outcomes solely through the exposure. Metabolites with IVW p-values below 0.05 underwent a Confounding analysis to identify any SNPs that violated the MR Hypothesis. To assess the association of IVs with known risk factors such as hypertension, hyperlipidemia, coronary artery disease, smoking, obesity, and diabetes, we examined the IVs for metabolites on the PhenoscannerV2 website (http://www.phenoscanner.medschl.cam.ac.uk/), a tool for exploring associations between genetic variants and phenotype. If any SNPs were found to be linked to these confounding factors (*p* < 1 × 10^−5^) (Supplementary Table S1), the MR analysis was repeated after excluding these SNPs to ensure the reliability of the results. To further investigate the nature of causal influence, we incorporated four additional MR models: MR-Egger regression, the weighted median method, the simple mode-based estimator, and the weighted mode-based estimator (Bowden et al., 2015).

### Sensitivity analysis

The diversity in experimental conditions, analytical platforms, and study subjects introduces potential heterogeneity in two-sample MR analyses, which can lead to biased estimations of causal effects. To address this, we employed the Cochran Q test was used to evaluate heterogeneity (Cohen et al., 2015), with a *p*-value below 0.05 indicating the presence of heterogeneity among the IVs. Conversely, a *p*-value greater than 0.05 suggests no evidence of heterogeneity, allowing us to disregard its impact on causal effect estimation.

When utilizing the IVW method to investigate causal relationships, unknown confounding factors can affect genetic multiplicity and introduce bias in causal effect estimation. To examine this, a horizontal pleiotropy test was conducted by analyzing the intercept of the MR-Egger regression and evaluating the corresponding *p*-value (Verbanck et al., 2018). If the intercept is close to 0 (<0.1) with a *p*-value greater than 0.05 indicates no proof of horizontal pleiotropy. Furthermore, the MR-PRESSO, a method for testing horizontal pleiotropy in Mendelian randomization studies, was employed to further evaluate horizontal pleiotropy and identify potential outliers.

Following the heterogeneity and horizontal pleiotropy tests, a sensitivity analysis was conducted on the qualified metabolites using the leave-one-out method (Nolte, 2020). This approach systematically removes each related SNP, calculates the overall effect by aggregating the remaining SNPs, and assesses the impact of each SNP on the metabolites. If the overall error line remains relatively stable after excluding each SNP (all error lines do not pass through 0), it indicates reliable results.

### Metabolic pathway and enrichment analysis

The analysis was performed through an online metabolomics data analysis website (https://www.metaboanalyst.ca/MetaboAnalyst/faces/home.xhtml), specifically utilizing the Enrichment Analysis and Pathway Analysis modules in the Annotated Features mode. We retrieved the corresponding IDs of these metabolites from the Human Metabolome Database (https://hmdb.ca/). Subsequently, we utilized these IDs to investigate pathways and enrichment using data from SMPDB (https://smpdb.ca/) and the KEGG database (https://www.kegg.jp/). This comprehensive approach enabled us to gather metabolite sets and pathways associated with CHD.

### Statistical analysis

LD analyses were conducted using PLINK software (version 1.9). We performed the LD analysis by selecting SNPs with *p* < 0.05, then filtering out those with a Minor Allele Frequency < 5% and missing genotypes > 5%. and ultimately, we selected SNPs with *r* ^2^ < 0.1 within a 500 kb distance.

Two-sample MR analyses, along with sensitivity analyses, were performed using the TwoSampleMR package (version 0.5.6) and the Gwasglue MR package (version 0.0.0.9000) in R (version 4.2.3).

## Results

### IV information

Among the SNPs exhibiting robust associations (*p* < 1 × 10^−5^; LD *r* ^2^ < 0.1 within 500 kb distance; F > 10) with 486 serum metabolites, 10,420 of these demonstrated an intriguing overlap with CHD. Notably, this overlap emerged after meticulously excluding the SNPs that displayed strong associations (*p* < 1 × 10^−5^) with CHD. SNPs associated with confounding factors (*p* < 1 × 10^−5^) were eliminated in subsequent analyses (Supplementary Table S1).

### MR analysis results

In this study, the IVW model served as the primary approach for estimating the causal relationships between blood metabolites and the risk of CHD (Supplementary Table S2). A total of 26 metabolites comprising 15 known metabolites and 11 unknown metabolites displayed a nominally significant relationship (*p* < 0.05, IVW method) with CHD risk (Table 1). The 15 known metabolites can be categorized into nine lipids (arachidonate (20:4n6), deoxycholate, carnitine, glycerophosphorylcholine, scyllo-inositol, 10-nonadecenoate (19:1n9), laurylcarnitine, tetradecanedioate, hexadecanedioate); three peptides (ADSGEGDFXAEGGGVR, pro-hydroxy-pro, X-14205--alpha-glutamyltyrosine); two energy substances (citrate, acetylphosphate) and one nucleotide (inosine).

**TABLE 1:** Metabolites significantly associated with CHD risk based on IVW results (*p* < 0.05).

ID	Metabolite	nSNP	Beta	SE	P	OR (95%CI)
Known Metabolites:						
M01110	arachidonate (20:4n6)	24	0.45	0.18	1.50E-02	1.56 (1.09-2.24)
M01114	deoxycholate	15	0.18	0.06	3.16E-03	1.20 (1.06-1.35)
M01123	inosine	13	-0.07	0.03	3.51E-02	0.93 (0.87-1.00)
M01564	citrate	48	0.29	0.15	4.74E-02	1.33 (1.00-1.78)
M15488	acetylphosphate	17	0.78	0.32	1.50E-02	2.18 (1.16-4.09)
M15500	carnitine	221	0.29	0.13	2.43E-02	1.33 (1.04-1.71)
M15990	glycerophosphorylcholine	18	-0.25	0.10	1.00E-02	0.78 (0.65-0.94)
M32379	scyllo-inositol	13	0.22	0.10	2.50E-02	1.24 (1.03-1.50)
M33084	ADSGEGDFXAEGGGVR*	8	-0.23	0.08	5.86E-03	0.80 (0.68-0.94)
M33972	10-nonadecenoate (19:1n9)	7	-0.44	0.18	1.50E-02	0.64 (0.45-0.92)
M34534	laurylcarnitine	13	0.29	0.11	7.43E-03	1.34 (1.08-1.66)
M35127	pro-hydroxy-pro	20	0.31	0.14	3.33E-02	1.36 (1.02-1.80)
M35669	tetradecanedioate	25	-0.15	0.06	1.82E-02	0.86 (0.76-0.97)
M35678	hexadecanedioate	27	-0.20	0.07	2.17E-03	0.82 (0.72-0.93)
M36131	X-14205--alpha-glutamyltyrosine	16	0.19	0.08	2.45E-02	1.20 (1.02-1.42)
Unkwon Metabolites:						
M12626	X-03003	9	0.58	0.28	4.07E-02	1.79 (1.02-3.13)
M32761	X-11444	17	0.38	0.11	3.79E-04	1.47 (1.19-1.82)
M32808	X-11491	17	-0.23	0.07	1.28E-03	0.79 (0.69-0.91)
M32846	X-11529	25	-0.13	0.03	1.37E-05	0.88 (0.83-0.93)
M32855	X-11538	24	-0.28	0.06	7.15E-06	0.76 (0.67-0.86)
M33359	X-12013	9	0.09	0.04	1.64E-02	1.10 (1.02-1.18)
M34221	X-12627	13	0.42	0.11	1.73E-04	1.53 (1.22-1.90)
M34327	X-12717	9	0.11	0.05	3.94E-02	1.12 (1.01-1.24)
M34453	X-12776	3	-1.52	0.56	6.41E-03	0.22 (0.07-0.65)
M35187	X-13429	12	-0.18	0.06	3.43E-03	0.83 (0.73-0.94)
M36552	X-14625	20	0.45	0.21	3.33E-02	1.57 (1.04-2.38)

Footnotes: **nSNP**, number of the SNP; **SE**, standard error; **OR**, odds ratio; **95% CI**, 95% confidence interval; **X**, unknown metabolite.

These known metabolites can be categorized into 9 risk molecules and 6 protective metabolites, as determined by their association with the risk of CHD using the IVW method (Table 2). Of these, inosine (OR = 0.93, 95% CI: 0.87–1.00), glycerophosphorylcholine (OR = 0.78, 95% CI: 0.65–0.94), ADSGEGDFXAEGGGVR* (OR = 0.80, 95% CI: 0.68–0.94), 10-nonadecenoate (19:1n9) (OR = 0.64, 95% CI: 0.45–0.92), tetradecanedioate (OR = 0.86, 95% CI: 0.76–0.97), and hexadecanedioate (OR = 0.82, 95% CI: 0.72–0.93) exhibited potentially reduced CHD risk (Table 2). Conversely, arachidonate (20:4n6) (OR = 1.56, 95% CI: 1.09–2.24), deoxycholate (OR = 1.20, 95% CI: 1.06–1.35), citrate (OR = 1.33, 95% CI: 1.00–1.78), acetylphosphate (OR = 2.18, 95% CI: 1.16–4.09), carnitine (OR = 1.33, 95%CI: 1.04–1.71), scyllo-inositol (OR = 1.24, 95% CI: 1.03–1.50), laurylcarnitine (OR = 1.34, 95%CI: 1.08–1.66), pro-hydroxy-pro (OR = 1.36, 95% CI: 1.02–1.80), and X-14205--alpha-glutamyltyrosine (OR = 1.20, 95% CI: 1.02–1.42) displayed potential increased CHD risk (Table 2).

**TABLE 2:** Five MR models investigating causal associations between 15 known metabolites and CHD risk, including heterogeneity and horizontal pleiotropy analyses.

Metabolite (nSNP)	Method	P	OR (95%CI)	P_Heter_	P_Horiz_
Arachidonate (20:4n6) (24)	MR Egger	7.77E-02	2.18 (0.95-4.97)	0.20	0.39
	Weighted median	1.62E-02	1.80 (1.11-2.90)		
	Inverse variance weighted	1.50E-02	1.56 (1.09-2.24)	0.21	
	Simple mode	9.61E-02	2.30 (0.90-5.89)		
	Weighted mode	7.08E-02	2.27 (0.97-5.30)		
Deoxycholate (15)	MR Egger	4.10E-01	1.12 (0.86-1.45)	0.93	0.58
	Weighted median	7.34E-02	1.15 (0.99-1.33)		
	Inverse variance weighted	3.16E-03	1.20 (1.06-1.35)	0.94	
	Simple mode	2.80E-01	1.16 (0.90-1.50)		
	Weighted mode	2.19E-01	1.16 (0.92-1.47)		
Inosine (13)	MR Egger	4.17E-01	0.95 (0.86-1.06)	0.43	0.58
	Weighted median	1.04E-01	0.92 (0.84-1.02)		
	Inverse variance weighted	3.51E-02	0.93 (0.87-1.00)	0.49	
	Simple mode	3.26E-01	0.92 (0.79-1.08)		
	Weighted mode	1.45E-01	0.92 (0.84-1.02)		
Citrate (48)	MR Egger	8.19E-02	1.97 (0.93-4.17)	0.88	0.27
	Weighted median	2.36E-01	1.27 (0.85-1.90)		
	Inverse variance weighted	4.74E-02	1.33 (1.00-1.78)	0.87	
	Simple mode	4.82E-01	1.36 (0.58-3.18)		
	Weighted mode	3.81E-01	1.36 (0.69-2.68)		
Acetylphosphate (17)	MR Egger	3.38E-01	2.98 (0.34-26.07)	0.34	0.77
	Weighted median	3.87E-01	1.47 (0.61-3.50)		
	Inverse variance weighted	1.50E-02	2.18 (1.16-4.09)	0.40	
	Simple mode	9.78E-01	0.98 (0.20-4.80)		
	Weighted mode	8.07E-01	1.19 (0.30-4.78)		
Carnitine (221)	MR Egger	5.68E-01	1.19 (0.65-2.17)	0.45	0.69
	Weighted median	1.47E-01	1.38 (0.89-2.12)		
	Inverse variance weighted	2.43E-02	1.33 (1.04-1.71)	0.47	
	Simple mode	3.98E-02	3.55 (1.07-11.82)		
	Weighted mode	9.45E-02	1.81 (0.91-3.63)		
Glycerophosphorylcholine (18)	MR Egger	2.37E-02	0.72 (0.55-0.93)	0.19	0.37
	Weighted median	1.89E-02	0.73 (0.57-0.95)		
	Inverse variance weighted	1.00E-02	0.78 (0.65-0.94)	0.19	
	Simple mode	3.12E-01	0.76 (0.45-1.28)		
	Weighted mode	1.98E-02	0.74 (0.58-0.93)		
scyllo-inositol (13)	MR Egger	2.24E-01	1.22 (0.90-1.65)	0.88	0.88
	Weighted median	3.09E-02	1.37 (1.03-1.82)		
	Inverse variance weighted	2.50E-02	1.24 (1.03-1.50)	0.92	
	Simple mode	1.32E-01	1.44 (0.93-2.23)		
	Weighted mode	8.51E-02	1.37 (0.99-1.90)		
ADSGEGDFXAEGGGVR* (8)	MR Egger	3.32E-01	0.81 (0.54-1.20)	0.75	0.95
	Weighted median	1.31E-02	0.76 (0.62-0.94)		
	Inverse variance weighted	5.86E-03	0.80 (0.68-0.94)	0.84	
	Simple mode	1.55E-01	0.77 (0.56-1.06)		
	Weighted mode	5.33E-02	0.78 (0.63-0.96)		
10-nonadecenoate (19:1n9) (7)	MR Egger	9.37E-01	1.04 (0.40–2.74)	0.43	0.34
	Weighted median	1.23E-01	0.69 (0.43–1.11)		
	Inverse variance weighted	1.50E-02	0.64 (0.45–0.92)	0.43	
	Simple mode	2.90E-01	0.64 (0.30–1.36)		
	Weighted mode	3.36E-01	0.69 (0.35–1.38)		
Laurylcarnitine (13)	MR Egger	6.54E-01	1.15 (0.64–2.04)	0.44	0.58
	Weighted median	1.46E-01	1.23 (0.93–1.64)		
	Inverse variance weighted	7.43E-03	1.34 (1.08–1.66)	0.50	
	Simple mode	6.56E-01	1.11 (0.71–1.72)		
	Weighted mode	5.05E-01	1.16 (0.76–1.79)		
Pro-hydroxy-pro (20)	MR Egger	8.09E-01	1.09 (0.56–2.11)	0.61	0.47
	Weighted median	9.06E-02	1.43 (0.95–2.16)		
	Inverse variance weighted	3.33E-02	1.36 (1.02–1.80)	0.64	
	Simple mode	2.04E-01	1.66 (0.78–3.54)		
	Weighted mode	1.83E-01	1.54 (0.83–2.86)		
Tetradecanedioate (25)	MR Egger	5.54E-02	0.79 (0.62–0.99)	0.12	0.39
	Weighted median	2.85E-01	0.91 (0.77–1.08)		
	Inverse variance weighted	1.82E-02	0.86 (0.76–0.97)	0.12	
	Simple mode	8.25E-01	1.04 (0.73–1.49)		
	Weighted mode	6.91E-01	0.95 (0.75–1.21)		
Hexadecanedioate (27)	MR Egger	5.60E-02	0.77 (0.60–0.99)	0.30	0.63
	Weighted median	5.00E-03	0.74 (0.60–0.91)		
	Inverse variance weighted	2.17E-03	0.82 (0.72–0.93)	0.34	
	Simple mode	3.28E-01	0.80 (0.51–1.24)		
	Weighted mode	2.22E-02	0.70 (0.53–0.93)		
X-14205--alpha -glutamyltyrosine (16)	MR Egger	3.74E-01	1.28 (0.75–2.19)	0.62	0.81
	Weighted median	4.24E-02	1.25 (1.01–1.54)		
	Inverse variance weighted	2.45E-02	1.20 (1.02–1.42)	0.69	
	Simple mode	7.18E-01	0.93 (0.62–1.39)		
	Weighted mode	7.57E-01	0.94 (0.63–1.39)		

Footnotes: **nSNP**, number of the SNP; **OR**, odds ratio; **95% CI**, 95% confidence interval; **P_Heter_**, P_Heterogeneity_; **P_Horiz_**, P_Horizontal pleiotropy_.

Subsequently, we employed four additional models (Bowden et al., 2015) to evaluate the causal effects between these metabolites and CHD risk (Table 2). Two exhibited significances in at least three MR models and consistently demonstrated causal effects across all models (Table 2; Supplementary Figure S1). The two metabolites are hexadecanedioate (P _IVW_ = 2.17 × 10^−3^, P _MR Egger_ = 5.60 × 10^−2^, P _Weighted median_ = 5.00 × 10^−3^, P _Simple mode_ = 3.28 × 10^−1^, P _Weighted mode_ = 2.22 × 10^−2^) (Table 2) and glycerophosphorylcholine (P _IVW_ = 1.00 × 10^−2^, P _MR Egger_ = 2.37 × 10^−2^, P _Weighted median_ = 1.89 × 10^−2^, P _Simple mode_ = 3.12 × 10^−1^, P _Weighted mode_ = 1.98 × 10^−2^) (Table 2). The results for hexadecanedioate and glycerophosphorylcholine were consistent across all five models (Supplementary Figures S1A, B). For hexadecanedioate, the estimated effect obtained from MR-Egger regression closely resembled that of IVW, with relatively wide confidence intervals (Supplementary Figure S1A). We observed the presence of a potential outlier in this case, whereas the funnel plot (Supplementary Figure S2) indicated an almost symmetrical distribution of data points when employing individual SNPs as instrumental variables (13 points versus 14 points).

### Assessment of the reliability and stability of the results

To ensure the validity of our findings, we undertook rigorous tests to evaluate the reliability and stability of the results concerning the known metabolites. The test results, employing MR-Egger and MR-PRESSO methods, demonstrated *p*-values above 0.05. Moreover, the intercept of MR-Egger regression was nearly 0 (<0.1), indicating the absence of heterogeneity and horizontal pleiotropy among these metabolites (Table 2 and Supplementary Table S3).

**FIGURE 2:**
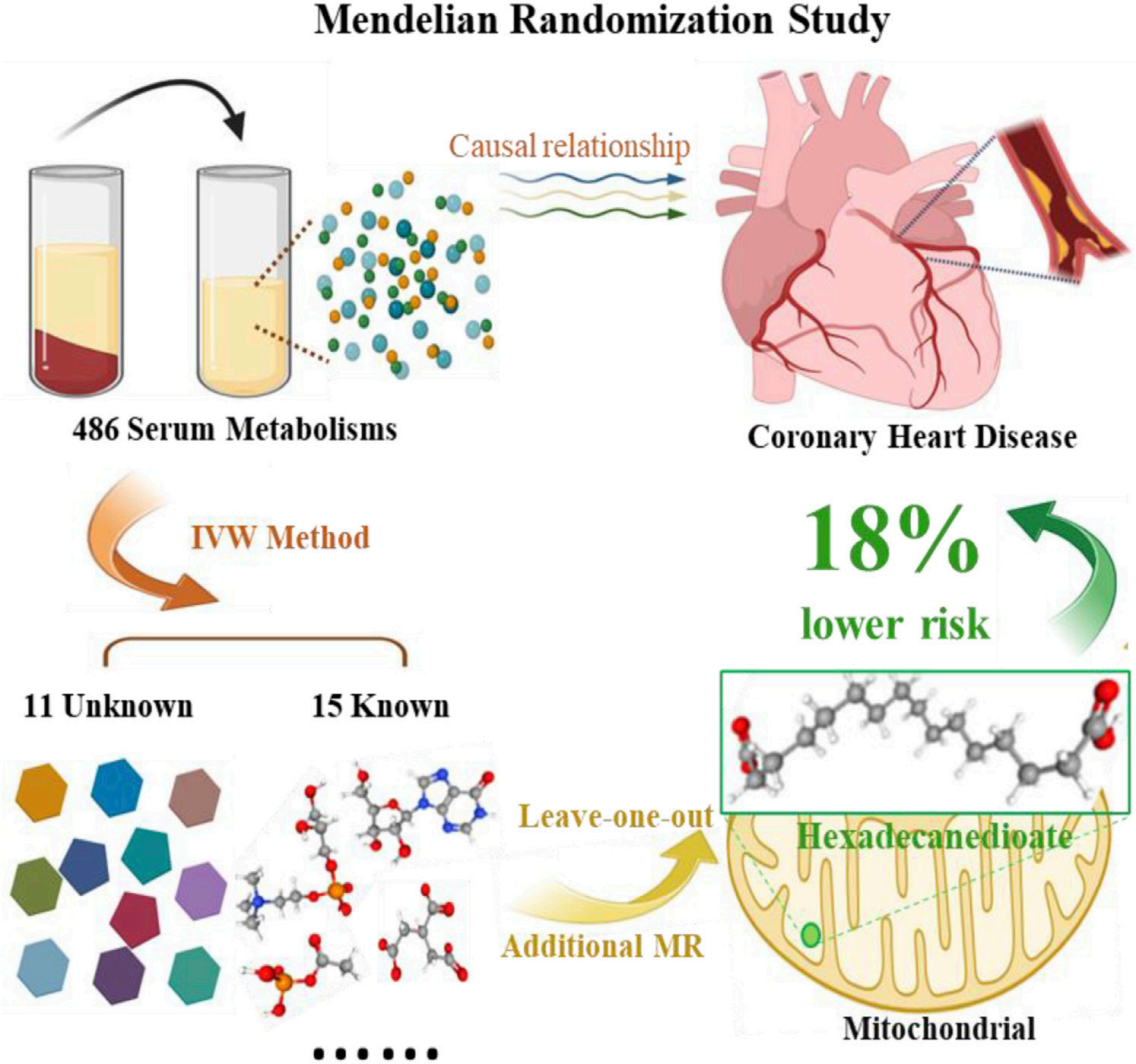
Graphical summary of the Mendelian Randomization Study.

Regarding the two metabolites, hexadecanedioate and glycerophosphorylcholine, which demonstrated notable robustness by displaying significant in at least three MR models, we conducted sensitivity analyses employing a leave-one-out approach to evaluate their stability. The results were not sensitive to the exclusion of individual SNPs associated with hexadecanedioate, suggesting a consistent and significant effect in reducing the risk of CHD by 18% (Table 2; Figure 2). However, it was observed that one instrumental variable (rs1978450) associated with glycerophosphorylcholine exerted a significant influence on the result (Supplementary Figures S1C, D). Consequently, after removing the rs1978450, we re-conducted MR analyses using the five models, only to find that the results were no longer significant (Supplementary Figure S1E).

### Metabolic pathway and enrichment analysis

In the pathway and enrichment analysis of the 15 known metabolites, we uncovered five metabolic pathways that exhibited relative significance (*p* < 0.1). The identified metabolic pathways encompassed “Ether lipid metabolism” (*p* = 0.0630), “Citrate cycle (TCA cycle)” (*p* = 0.0630), and “Pyruvate metabolism” (*p* = 0.0691), “Alanine, aspartate and glutamate metabolism” (*p* = 0.0872) and “Glyoxylate and dicarboxylate metabolism” (*p* = 0.9917) (Table 3; Supplementary Figure S3, and Supplementary Tables S4, S5). Additionally, the enrichment analysis identified significant metabolite sets, including “Citrate cycle (TCA cycle)” (*p* = 0.0757), “Ether lipid metabolism” (*p* = 0.0757), “Pyruvate metabolism” (*p* = 0.083), and “Lysine degradation (*p* = 0.0939) (Table 3; Supplementary Figures S4, S5, and Supplementary Table S6). Notably, all these metabolic pathways are closely associated with, or take place within, mitochondrial function.

**TABLE 3:** Pathway and enrichment analysis results for CHD-Related metabolites.

Metabolic pathway	Involved metabolites	Pvalue	Database	Associated organelles
Pathway Analysis				
Ether lipid metabolism	Glycerophosphorylcholine	0.062952	KEGG	Mitochondrial
Citrate cycle (TCA cycle)	Citrate	0.062952	KEGG SMPDB	Mitochondrial
Pyruvate metabolism	Acetyl phosphate	0.069068	KEGG SMPDB	Mitochondrial
Alanine, aspartate and glutamate metabolism	Citrate	0.087226	KEGG SMPDB	Mitochondrial
Glyoxylate and dicarboxylate metabolism	Citrate	0.099173	KEGG	Mitochondrial
Enrichment Analysis				
Citrate cycle (TCA cycle)	Citric acid	0.0757	KEGG	Mitochondrial
Ether lipid metabolism	Glycerophosphorylcholine	0.0757	KEGG	Mitochondrial
Pyruvate metabolism	Acetyl phosphate	0.083	KEGG	Mitochondrial
Lysine degradation	L-Carnitine	0.0939	KEGG	Mitochondrial

## Discussion

In this study, we conducted an unbiased two-sample MR analysis to investigate the causal relationship between 486 blood metabolites and the risk of CHD. To ensure the utmost reliability, we collected the largest GWAS and expansive CHD GWAS summary data from public databases carefully. Utilizing genetic variants as IVs, we identified 15 known and 11 unknown metabolites that exhibited potential as predictors of CHD risk, as confirmed by our primary IVW analysis. These known metabolites can be classified into six protective factors (inosine, glycerophosphorylcholine, ADSGEGDFXAEGGGVR, 10-nonadecenoate, tetradecanedioate, hexadecanedioate) and nine risk factors (arachidonate (20:4n6), deoxycholate, citrate, acetylphosphate, carnitine, scyllo-inositol, laurylcarnitine, pro-hydroxy-pro, X-14205--alpha-glutamyltyrosine). Notably, a substantial number of these known metabolites are associated with the energy-producing function of mitochondria.

To delve deeper into the underlying biological mechanisms by which these metabolites impact the CHD development, we conducted comprehensive metabolic pathways and enrichment analyses of the 15 identified metabolites. The results unveiled significant enrichment in certain signaling pathways closely linked to mitochondrial energy metabolism, such as “Ether lipid metabolism”, “Citrate cycle”, and others. These findings strongly suggest that aberrant mitochondrial function may play a pivotal role in the occurrence and progression of CHD.

To further enhance the reliability and stability of our findings, we conducted additional MR models. The results consistently affirmed the association of hexadecanedioate and glycerophosphorylcholine with a decreased risk of CHD in at least three MR models. Glycerophosphorylcholine, a crucial component of phospholipids contributing to the structural integrity of cell membranes (Sonkar et al., 2019), exerts an impact on the efficiency of mitochondrial respiration and oxidative phosphorylation - the processes responsible for ATP synthesis within mitochondria (Modica-Napolitano and Renshaw, 2004). Regrettably, it is essential our study’s analysis of glycerophosphorylcholine did not withstand the final leave-one-out analysis, necessitating caution in interpreting its causal relationship with CHD. Regarding hexadecanedioate, also known as palmitate, it possesses a distinctive structural composition, characterized by a sixteen-carbon alkyl chain and two carboxyl groups (Menni et al., 2015). It plays a crucial role in the mitochondrial fatty acid oxidation pathway, where long-chain fatty acids are broken down to produce energy for the cell (Matsunaga et al., 2000). Emerging studies have indicated that hexadecanedioate may impact mitochondrial function and contribute to mitochondrial dysfunction, which is associated with oxidative stress through ROS/GSH/GPX4 pathway, inflammatory responses, and endothelial dysfunction (Menni et al., 2017), (Peoples et al., 2019). Our groundbreaking findings illuminated a compelling revelation: hexadecanedioate exhibited a significant association with an 18% reduction in the incidence of CHD.

### Innovations and limitations

Our study presents several innovative aspects that contribute significantly to the field. Firstly, we adopt a molecular mechanism approach by considering blood metabolites as exposure factors, providing a robust theoretical foundation and significant clinical research value in exploring the causal relationships between metabolites and the risk of CHD. Secondly, we uphold the highest standards of quality control, implementing rigorous measures, and employing diverse analysis methods, including multiple models, to thoroughly evaluate the causal effects. As a result, the findings of our study can be considered reliable and stable. Thirdly, unlike previous MR analyses that focus on individual exposure factors, the comprehensive analysis of a large number of blood metabolites presents extensive workloads and analytical challenges. The analysis strategy we propose offers valuable insights for similar studies in the field.

However, we acknowledge certain limitations in the study. To begin with, half of the CHD risk predictors identified in our preliminary analysis (solely employing the IVW method) are unidentified metabolites with uncertain functional structures, which may require professional chemists to verify the formula based on mass spectrometry results in the future. Therefore, our study’s findings are limited by these uncertainties. Moreover, the population exposed to our study comprises individuals of European descent; with approximately 77% of the outcome population being European and a small representation of non-European individuals, this demographic imbalance may impact the generalizability of our findings to other ethnic groups, we utilized the ancestry-specific principal components and adjustments from the original dataset to control for population stratification. This approach helps ensure that our results are not biased by genetic structure differences across the different ancestry groups. Additionally, although we observed a nominal causal association between hexadecanedioate and CHD using an unbiased two-sample MR approach, it is essential to recognize that this relationship remains theoretical, pending further mechanistic validation. Therefore, further investigation is still needed to clarify the role of hexadecanedioate in CHD pathogenesis and establish a definitive confirmation of this causal relationship. While we have taken steps to minimize confounding—such as adjusting for known confounders and applying MR methods that are less susceptible to confounding than traditional observational studies—residual confounding may still arise due to unmeasured or unknown factors. As we embrace these limitations as opportunities for growth, our study provides a foundation for future research endeavors, contributing to the advancement of knowledge and understanding in the realm of CHD and its molecular underpinnings.

### Conclusion

In summary, our study employed a rigorous two-sample MR approach to unravel the causal relationships between 486 blood metabolites and CHD risk in an extensive cohort of over 0.12 million individuals of European descent. Through meticulous analysis, we unveiled a total of 26 serum metabolites linked to CHD, encompassing 6 protective metabolites, nine risk factors and 11 previously unidentified metabolites, many of which are known to be associated with mitochondrial function. Our pathway and enrichment analysis further revealed significant pathways related to mitochondrial function, such as the ‘Citrate cycle’, among others. Remarkably, our findings indicated that hexadecanedioate, a palmitoyl lipid located in and metabolized in mitochondria, could nominally reduce the risk of CHD by 18%. These groundbreaking discoveries substantially enhance our understanding of the intricate interplay between blood metabolites and CHD, suggesting that these serum metabolites may exert their influence on the development of CHD by modulating mitochondrial function. This revelation opens up broad prospects for developing personalized explanations or markers that can shed light on the underlying biological changes in disease states. By unraveling these vital connections, our study lays the foundation for future research to advance the prevention, diagnosis, and management of CHD.

## Data Availability

Existing datasets are available in a publicly accessible repository: Publicly available datasets were analyzed in this study. This data can be found here: the Metabolomics GWAS Server (https://metabolomics.helmholtz-muenchen.de/gwas/) for metabolomics data, and the CHD Datasets (https://gwas.mrcieu.ac.uk/) for CHD data.

## References

[B1] Borges M. C. Haycock P. C. Zheng J. Hemani G. Holmes M. V. Davey Smith G. (2022). Role of circulating polyunsaturated fatty acids on cardiovascular diseases risk: analysis using Mendelian randomization and fatty acid genetic association data from over 114,000 UK Biobank participants. BMC Med. 20, 210. 10.1186/s12916-022-02399-w 35692035 PMC9190170

[B2] Bowden J. Davey Smith G. Burgess S. (2015). Mendelian randomization with invalid instruments: effect estimation and bias detection through Egger regression. Int. J. Epidemiol. 44, 512–525. 10.1093/ije/dyv080 26050253 PMC4469799

[B3] Burgess S. Thompson S. G. Collaboration C. C. G. (2011). Avoiding bias from weak instruments in Mendelian randomization studies. Int. J. Epidemiol. 40, 755–764. 10.1093/ije/dyr036 21414999

[B4] Cohen J. F. Chalumeau M. Cohen R. Korevaar D. A. Khoshnood B. Bossuyt P. M. (2015). Cochran's Q test was useful to assess heterogeneity in likelihood ratios in studies of diagnostic accuracy. J. Clin. Epidemiol. 68, 299–306. 10.1016/j.jclinepi.2014.09.005 25441698

[B5] Dudbridge F. (2021). Polygenic mendelian randomization. Cold Spring Harb. Perspect. Med. 11, a039586. 10.1101/cshperspect.a039586 32229610 PMC7849343

[B6] Emdin C. A. Khera A. V. Kathiresan S. (2017). Mendelian randomization. Jama. 318, 1925–1926. 10.1001/jama.2017.17219 29164242

[B7] Goff D. C. Jr. Khan S. S. Lloyd-Jones D. Arnett D. K. Carnethon M. R. Labarthe D. R. (2021). Bending the curve in cardiovascular disease mortality: bethesda + 40 and beyond. Circulation 143, 837–851. 10.1161/CIRCULATIONAHA.120.046501 33617315 PMC7905830

[B8] Henderson A. (1996). Coronary heart disease: overview. Lancet London, Engl. 348 (Suppl. 1), s1–s4. 10.1016/s0140-6736(96)98001-0 8918518

[B9] Li J. Guasch-Ferre M. Chung W. Ruiz-Canela M. Toledo E. Corella D. (2020). The Mediterranean diet, plasma metabolome, and cardiovascular disease risk. Eur. Heart J. 41, 2645–2656. 10.1093/eurheartj/ehaa209 32406924 PMC7377580

[B10] Matsunaga I. Sumimoto T. Ueda A. Kusunose E. Ichihara K. (2000). Fatty acid-specific, regiospecific, and stereospecific hydroxylation by cytochrome P450 (CYP152B1) from Sphingomonas paucimobilis: substrate structure required for alpha-hydroxylation. Lipids 35, 365–371. 10.1007/s11745-000-533-y 10858020

[B11] Menni C. Graham D. Kastenmuller G. Alharbi N. H. Alsanosi S. M. McBride M. (2015). Metabolomic identification of a novel pathway of blood pressure regulation involving hexadecanedioate. Hypertension 66, 422–429. 10.1161/HYPERTENSIONAHA.115.05544 26034203 PMC4490909

[B12] Menni C. Metrustry S. J. Ehret G. Dominiczak A. F. Chowienczyk P. Spector T. D. (2017). Molecular pathways associated with blood pressure and hexadecanedioate levels. PloS one 12, e0175479. 10.1371/journal.pone.0175479 28403188 PMC5389832

[B13] Mirkov S. Myers J. L. Ramirez J. Liu W. (2012). SNPs affecting serum metabolomic traits may regulate gene transcription and lipid accumulation in the liver. Metabolism 61, 1523–1527. 10.1016/j.metabol.2012.05.004 22738862 PMC3867007

[B14] Modica-Napolitano J. S. Renshaw P. F. (2004). Ethanolamine and phosphoethanolamine inhibit mitochondrial function *in vitro*: implications for mitochondrial dysfunction hypothesis in depression and bipolar disorder. Biol. psychiatry 55, 273–277. 10.1016/s0006-3223(03)00784-4 14744468

[B15] Nolte I. M. (2020). Metasubtract: an R-package to analytically produce leave-one-out meta-analysis GWAS summary statistics. Bioinformatics 36, 4521–4522. 10.1093/bioinformatics/btaa570 32696040 PMC7750933

[B16] Peoples J. N. Saraf A. Ghazal N. Pham T. T. Kwong J. Q. (2019). Mitochondrial dysfunction and oxidative stress in heart disease. Exp. Mol. Med. 51 (12), 1–13. 10.1038/s12276-019-0355-7 PMC6923355 31857574

[B17] Sanna S. van Zuydam N. R. Mahajan A. Kurilshikov A. Vich Vila A. Vosa U. (2019). Causal relationships among the gut microbiome, short-chain fatty acids and metabolic diseases. Nat. Genet. 51, 600–605. 10.1038/s41588-019-0350-x 30778224 PMC6441384

[B18] Sekula P. Del Greco M. F. Pattaro C. Kottgen A. (2016). Mendelian randomization as an approach to assess causality using observational data. J. Am. Soc. Nephrol. 27, 3253–3265. 10.1681/ASN.2016010098 27486138 PMC5084898

[B19] Shin S. Y. Fauman E. B. Petersen A. K. Krumsiek J. Santos R. Huang J. (2014). An atlas of genetic influences on human blood metabolites. Nat. Genet. 46, 543–550. 10.1038/ng.2982 24816252 PMC4064254

[B20] Sonkar K. Ayyappan V. Tressler C. M. Adelaja O. Cai R. Cheng M. (2019). Focus on the glycerophosphocholine pathway in choline phospholipid metabolism of cancer. NMR Biomed. 32, e4112. 10.1002/nbm.4112 31184789 PMC6803034

[B21] Talmor-Barkan Y. Bar N. Shaul A. A. Shahaf N. Godneva A. Bussi Y. (2022). Metabolomic and microbiome profiling reveals personalized risk factors for coronary artery disease. Nat. Med. 28, 295–302. 10.1038/s41591-022-01686-6 35177859 PMC12365913

[B22] Tsepilov Y. A. Shin S.-Y. Soranzo N. Spector T. D. Prehn C. Adamski J. (2015). Nonadditive effects of genes in human metabolomics. Genetics 200, 707–718. 10.1534/genetics.115.175760 25977471 PMC4512538

[B23] Vasishta S. Ganesh K. Umakanth S. Joshi M. B. (2022). Ethnic disparities attributed to the manifestation in and response to type 2 diabetes: insights from metabolomics. Metabolomics 18, 45. 10.1007/s11306-022-01905-8 35763080 PMC9239976

[B24] Verbanck M. Chen C. Y. Neale B. Do R. (2018). Detection of widespread horizontal pleiotropy in causal relationships inferred from Mendelian randomization between complex traits and diseases. Nat. Genet. 50, 693–698. 10.1038/s41588-018-0099-7 29686387 PMC6083837

[B25] Zhu W. Li X. (2023). Liquid biopsy in coronary heart disease. Methods Mol. Biol. 2695, 279–293. 10.1007/978-1-0716-3346-5_19 37450126

[B26] Zuber V. Grinberg N. F. Gill D. Manipur I. Slob E. A. W. Patel A. (2022). Combining evidence from Mendelian randomization and colocalization: review and comparison of approaches. Am. J. Hum. Genet. 109, 767–782. 10.1016/j.ajhg.2022.04.001 35452592 PMC7612737

