# Supplementary material for: Causal relationships between serum metabolites and coronary heart disease risk: a mendelian randomization study
Source: Front Genet. 2025 Mar 20;16:1440364. doi: 10.3389/fgene.2025.1440364 (PMC11965349; doi:10.3389/fgene.2025.1440364)
Supplement: Supplementary file 2 [file Image1.pdf]

Causal relationships between serum metabolites and coronary heart disease risk: a mendelian randomization study

Supplementary Figures

Supplementary Figure S1 | Causal associations of hexadecanedioate and glycerophosphorylcholine with CHD and their sensitivity analyses.

A, B: Scatter plots depicting the 5 MR models (light blue line represents inverse variance weighted; blue line corresponds to MR Egger; light green line indicates the simple model-based estimator; green line shows the weighted median estimator; and red line represents the weighted model-based estimator) for hexadecanedioate (**A**) and glycerophosphorylcholine (**B**) with the risk of CHD. **C, D**: Forest plots displaying the results of leave-one-out analyses of the two metabolites, hexadecanedioate (**C**) and glycerophosphorylcholine (**D**), respectively. **E**: Re-analyses results of five MR models after removing of sensitive SNP (rs1978450) for glycerophosphorylcholine.

Supplementary Figure S2 | Funnel plot of Two-sample MR analysis based on the IVW and MR-Egger models with hexadecanedioate.

Supplementary Figure S3 | Pathway analysis of CHD-related metabolites.

Supplementary Figure S4 | SMPDB pathway enrichment of CHD-related metabolites.

Supplementary Figure S5 | KEGG pathway enrichment of CHD-related metabolites.

Supplementary Figure S1 | Causal associations of hexadecanedioate and glycerophosphorylcholine with CHD and their sensitivity analyses.

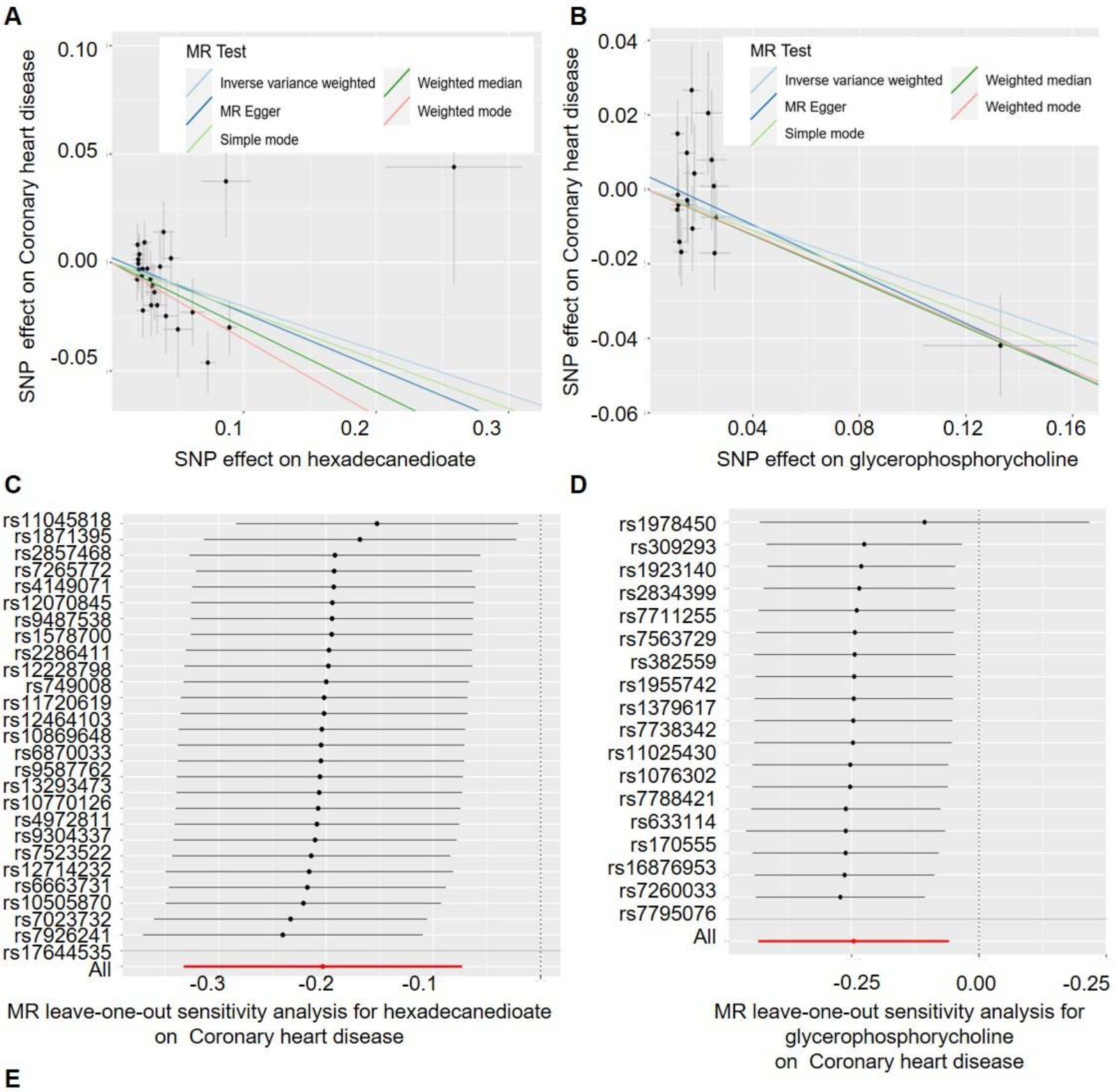

E

Metabolite (ID)	Method	nSNP	P	OR (95%CI)
Glycerophosphorycholine (M15990)	MR Egger	17	8.57E-01	0.90(0.30-2.69)
	Weighted median	17	2.12E-01	0.77(0.52-1.16)
	Inverse variance weighted	17	5.18E-01	0.90(0.65-1.24)
	Simple mode	17	4.56E-01	0.77(0.39-1.5)
	Weighted mode	17	4.06E-01	0.78(0.44-1.38)

Supplementary Figure S2 | Funnel plot of Two-sample MR analysis based on the IVW and MR-Egger models with hexadecanedioate.

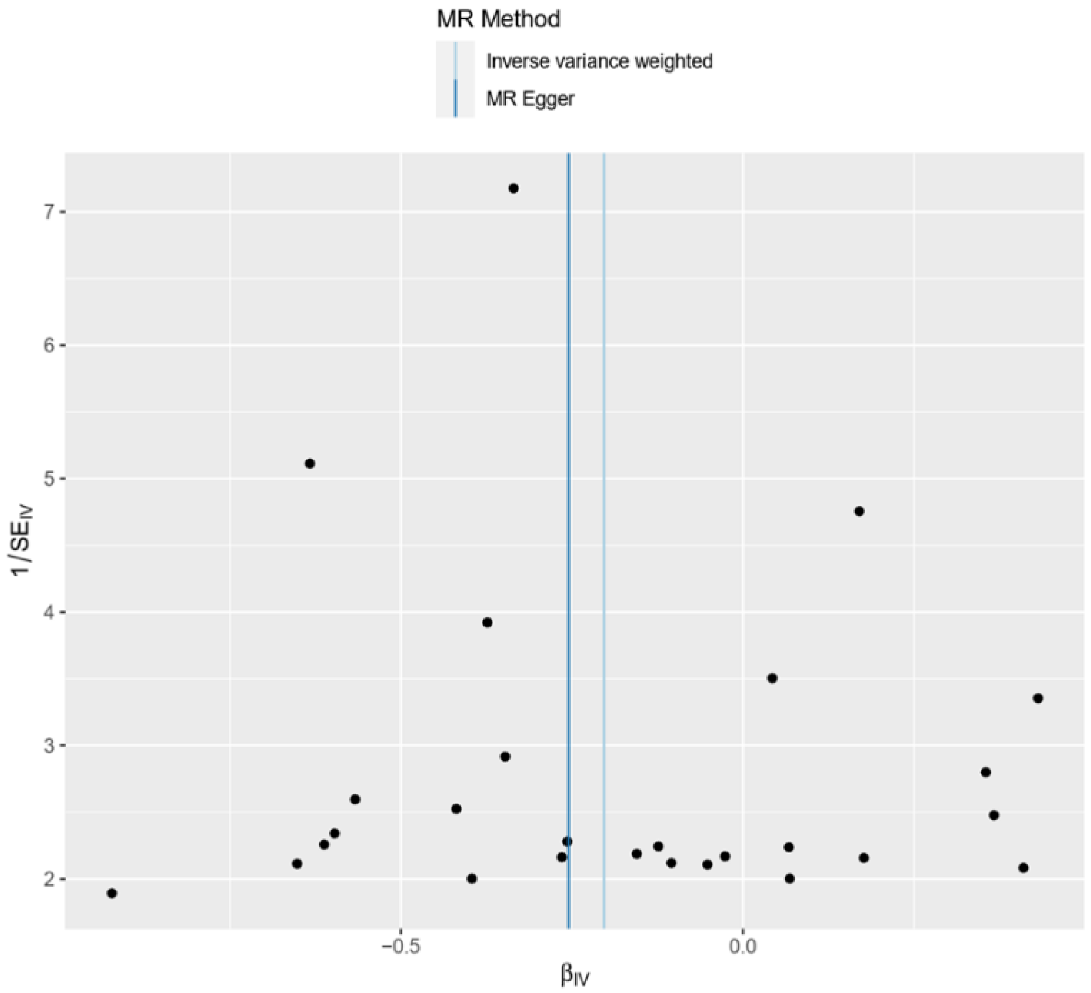

Supplementary Figure S3 | Pathway analysis of CHD-related metabolites.

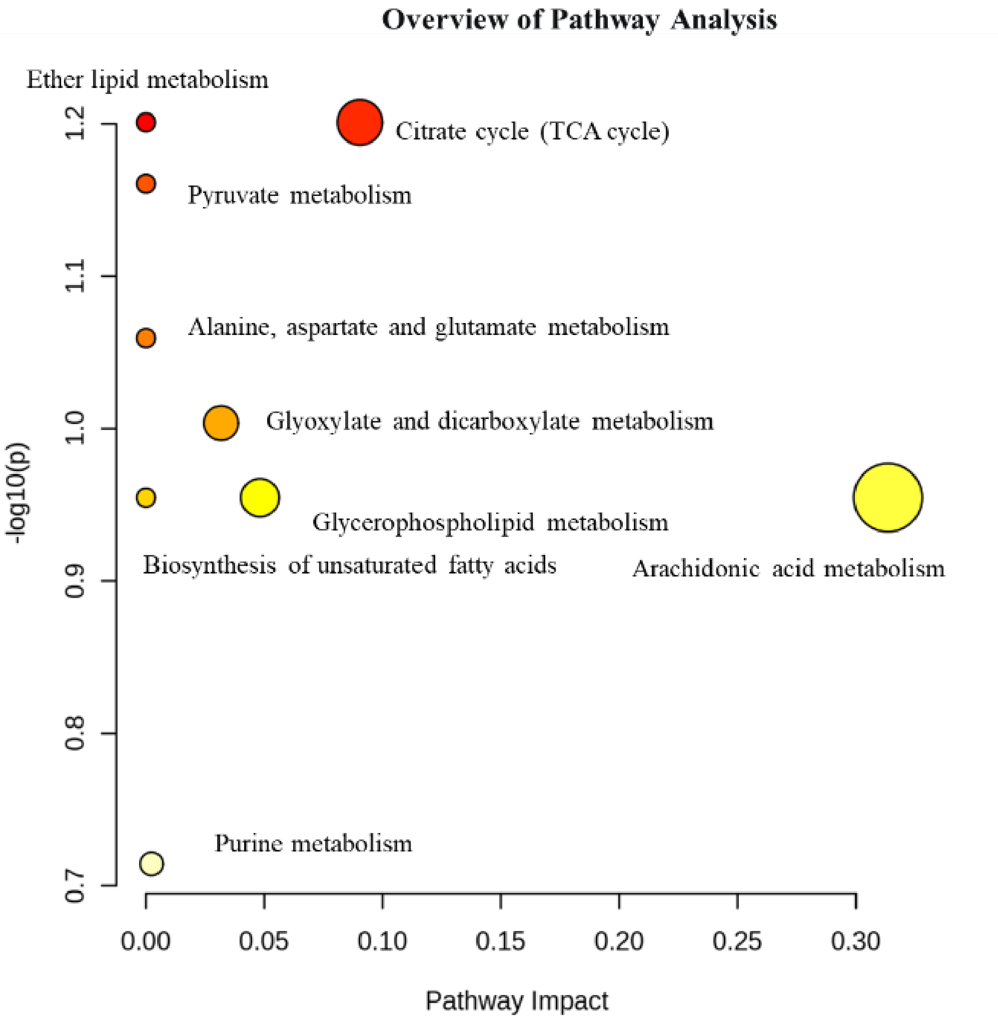

The metabolome view shows all matched pathways based on the *p*-values from the pathway enrichment analysis and pathway impact values from the pathway topology analysis.

Supplementary Figure S4 | SMPDB pathway enrichment of CHD-related metabolites.

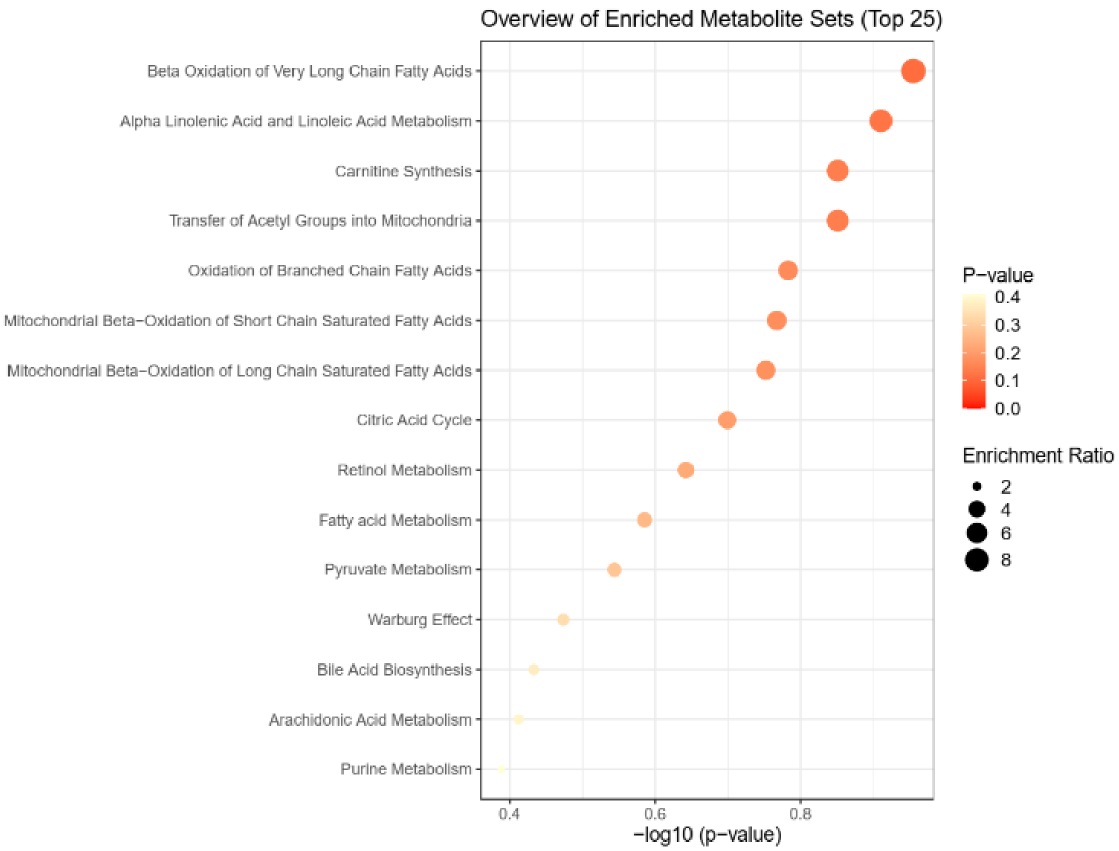

This figure shows the top 15 items.

Supplementary Figure S5 | KEGG pathway enrichment of CHD-related metabolites.

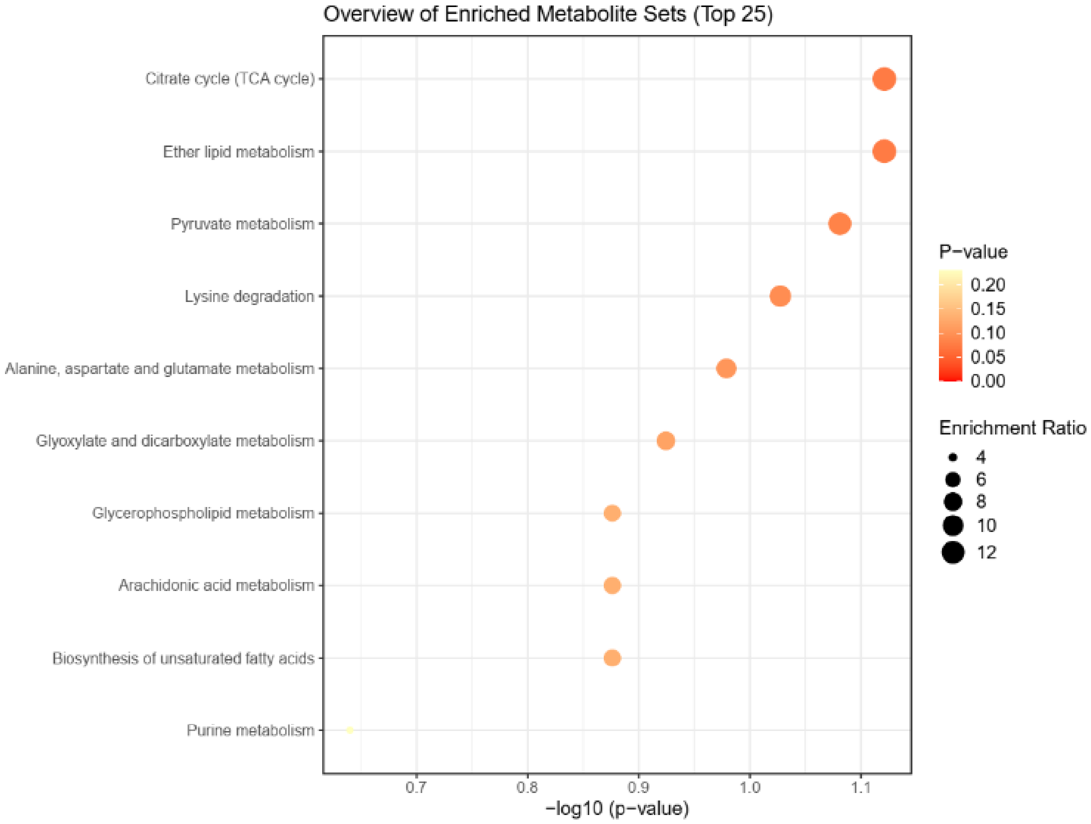

This figure shows the top 10 items.
